# Gastrodin induced HO-1 and Nrf2 up-regulation to alleviate H_2_O_2_-induced oxidative stress in mouse liver sinusoidal endothelial cells through p38 MAPK phosphorylation

**DOI:** 10.1590/1414-431X20187439

**Published:** 2018-08-16

**Authors:** Hongbin Zhang, Bo Yuan, Hanfei Huang, Siming Qu, Shikun Yang, Zhong Zeng

**Affiliations:** 1 Centre of Organ and Tissue Transplantation, the First Affiliated Hospital, Kunming Medical University, Kunming, Yunnan, China; 2 Department of Oncology, the First Affiliated Hospital, Kunming Medical University, Kunming, Yunnan, China

**Keywords:** Gastrodin, Liver sinusoidal endothelial cells, Heme oxygenase 1, Oxidative stress, Hydrogen peroxide

## Abstract

Nuclear factor erythroid-related factor 2 (Nrf2) has been implicated in several detoxifying and antioxidant defense processes. Nrf2-mediated heme oxygenase-1 (HO-1) expression was demonstrated to play a key role against oxidative stress. Gastrodin (GSTD) is a well-known active compound isolated from the roots of *Rhizoma gastrodiae*, a plant used in ancient Chinese traditional medicine. The aim of this work was to investigate whether GSTD could alleviate H_2_O_2_-induced oxidative stress in mouse liver sinusoidal endothelial cells (LSECs). In LSECs exposed to 1 mM H_2_O_2_, treatment with GSTD (1, 10, or 50 µM) resulted in higher cell viability than the untreated control. Treated cells maintained a higher Bcl2/Bax ratio and suppressed caspase-9 expression compared with untreated cells, reducing cell apoptosis. GSTD was protective for H_2_O_2_-induced oxidative injury by reducing the generation of intracellular reactive oxygen species and malondialdehyde. HO-1 and Nrf2 expressions were synergistically upregulated by GSTD. Inhibition of HO-1 by 10 µM zinc protoporphyrin resulted in less protective effects on cell viability and malondialdehyde reduction by GSTD treatment in H_2_O_2_-exposed LSECs. Additionally, phosphorylated p38 in LSECs exposed to H_2_O_2_ was elevated by GSTD. Inhibition of p38 phosphorylation by SB203580 did not induce Nrf2 and HO-1 expression after 1 or 10 µM GSTD treatment and the protective effect on cell viability and malondialdehyde reduction in H_2_O_2_-exposed LSECs was reduced. The data conclusively demonstrated that GSTD-induced HO-1 and Nrf2 expression is involved in protection of LSECs from H_2_O_2_-induced oxidative injury, which may be regulated by p38 phosphorylation.

## Introduction

Gastrodin (GSTD) is an active compound isolated from the roots of a plant used in ancient Chinese traditional medicine, *Rhizoma gastrodiae*, more commonly known as Tianma. Tianma is traditionally used to treat ailments such as headache, dizziness, convulsion, paralysis, rheumatism, and lumbago (1,2). Several formulations of GSTD (injection, tablet, and capsule) have been developed and are used clinically to treat dizziness and apoplexy in Southeast Asian countries (1). In recent decades, GSTD was found to be effective against Parkinson's and Alzheimer's diseases in animal models (3,4). GSTD was also found to be effective against several types of liver disease, including drug- and non-drug-induced liver injury, nonalcoholic fatty liver disease (NAFLD), and hepatic fibrosis (5–7). These studies indicated that GSTD protected liver cells as the disease progressed. Clinically, we found that using GSTD facilitated the recovery of patients subjected to liver transplantation (data not shown). Ischemia-reperfusion (IR) injury is common at the site of liver, heart, and limb transplantation (8). Inflammatory response and oxidative stress due to reactive oxygen species (ROS) accumulation are two main aspects associated with IR injury (9). IR injury could be alleviated if the severe inflammatory response and oxidative stress could be reduced. In line with the current understanding of IR, our study revealed that efficacy of GSTD treatment was mainly attributed to antioxidant and anti-inflammatory activities. GSTD treatment may help patients with liver disease recover more rapidly through the antioxidant and anti-inflammatory properties of the compound.

Liver sinusoidal endothelial cells (LSECs) are highly specialized endothelial cells, representing the interface between blood cells and hepatocytes/hepatic stellate cells (10). Approximately 15 to 20% of liver cells consist of LSECs, which form the wall of the liver and are the initial target of injury for some hepatotoxic drugs and toxins (10,11). LESCs are also susceptible to IR injury (12). Under physiological conditions, LSECs regulate hepatic vascular tone, maintaining the low portal pressure produced as a result of hepatic blood flow (10). In pathological conditions, LSECs play a key role in the initiation and progression of chronic liver diseases. They generally become capillarized and lose their protective properties, thus promoting angiogenesis and vasoconstriction (13,14). LSECs are implicated in liver regeneration following acute liver injury or partial hepatectomy because they are able to renew from LSEC progenitors (10). They sense changes in shear stress resulting from surgery, interacting with platelets and inflammatory cells. LSECs become damaged and undergo cell death after IR causes marked microcirculatory disturbances, leukocyte and platelet adhesion, diminished blood flow, and continuation of the ischemic process, leading to massive hepatic necrosis (15,16).

Nuclear factor erythroid-related factor 2 (Nrf2) is a transcription factor responsible for the regulation of cellular redox balance and protective antioxidant and phase II detoxification responses in mammals (17,18). One of the genes regulated through Nrf2 is heme oxygenase-1 (*HO-1*). HO-1/Nrf2 have been shown to be involved in the protection of burn-induced hepatic oxidative injury, carbon tetrachloride-induced liver injury, nickel-induced DNA methylation, and inflammation (19,20). Nrf2 also plays a critical role in the mechanism of hepatic IR injury and is considered a potential therapeutic target for preventing hepatic IR injury during liver surgery (21). In an IR animal model, activating transcription factor 3-mediated Nrf2/HO-1 signaling was shown to regulate toll-like receptor 4-driven inflammatory responses in livers (22). Brg1-mediated Nrf2/HO-1 pathway activation was also reported to reduce oxidative stress and alleviate hepatic IR injury (23). The aim of this work was to investigate the effects of GSTD treatment on H_2_O_2_-induced oxidative stress in mouse LSECs. The effects of GSTD treatment on cell apoptosis and viability, ROS generation, and malondialdehyde (MDA) content in H_2_O_2_-exposed LSECs were evaluated. Changes in HO-1 and Nrf2 expression induced by GSTD treatment in H_2_O_2_-exposed LSECs were studied. The mechanism of GSTD-regulated Nrf2/HO-1 expression to alleviate oxidative stress of LESCs is discussed.

## Material and Methods

### Cell culture

C57BL/6 mice (6-months old) were purchased from Procell Biotechnology Co. Ltd. (China). LSECs were isolated from mouse livers and maintained in Roswell Park Memorial Institute (RPMI) 1640 medium supplemented with 10% fetal calf serum and 1% penicillin/streptomycin. The cells were cultured in a humidified incubator under a 5% CO_2_ and 95% air atmosphere at 37°C.

### Oxidative stress induction

To induce oxidative stress, a stock solution of H_2_O_2_ (36%, Sigma, USA) was added directly to the culture medium to a final concentration of 1 mM. After 120 min of H_2_O_2_ exposure, a stock solution of 1 mM GSTD (purity>99%, dissolved in DMSO, Kunming Pharmaceutical Factory, China) was added to the culture medium to a final concentration of 1, 10, or 50 µM. When necessary, 1 µM (final concentration) of zinc protoporphyrin (Znpp, Sigma) or 10 µM (final concentration) of SB 203580 (Sigma) was added to the culture at the same time as GSTD addition. Unless otherwise specified, cells were cultured for 8 h after addition of GSTD. All the concentrations of the compounds were defined in preliminary experiments in our laboratory.

### Cell viability assay

LSECs (4×10^4^/well) were seeded and cultured in a 96-well dish containing RPMI 1640 with 10% FCS. The culture was then mixed with 50 µL RPMI 1640 and 5 µL Cell Counting Kit-8 (CCK-8; Dojindo, Japan) solution for 3 h. Absorbance at 450 nm was measured using a microplate reader (Model 680, BioRad Laboratories, USA).

### Cell apoptosis assay

Flow cytometry was performed following the manufacturer's instructions (BD Biosciences, USA). Cells were harvested then resuspended in 1× binding buffer at 1×10^5^ cells/mL. Next, 50 µL of the cell suspension, 5 µL annexin V-PE, and 5 µL 7-amino-actinomycin D were mixed together. After 20 min incubation in the dark, 400 µL of 1× binding buffer was added to the mixture. The rate of cellular apoptosis was measured using a FACSCalibur flow cytometer (BD Biosciences).

### Western blot

LSECs were lysed with RIPA lysis solution (DSL, USA). Total protein was extracted and then quantified using a BCA protein assay kit (Pierce, USA). Proteins were mixed with 4× loading buffer (Beyotime, China) and boiled for 5 min. A total of 20 µg protein was loaded onto a 12% sodium dodecyl sulfate polyacrylamide gel electrophoresis (SDS-PAGE) gel, subjected to electrophoresis, and then transferred to a polyvinylidene difluoride (PVDF, 0.45 µm, Sangon, China) membrane. The membrane was blocked in a solution of 5% fat-free milk and incubated for 30 min at room temperature. Next, the membrane was incubated with primary antibodies (1:6000, Abcam, USA) at 4°C overnight. Finally, the membrane was washed twice using phosphate buffer saline (PBS, pH=7.2) and incubated with secondary antibodies (1:4000, Abcam) at room temperature for 2 h. Bands were visualized using an enhanced chemiluminescence system (ECL, USA).

### MDA measurements

MDA, an end-product of lipid peroxidation, was measured in endothelial cell suspensions (5×10^6^/mL) using the thiobarbituric assay. MDA bis(dimethyl acetal) was used as a standard.

### ROS assay

Intracellular ROS was measured using a ROS assay kit (Sangon) containing a fluorescent probe (2′,7′-dichlorofluorescein diacetate (DCFH-DA). LSECs were incubated for 8 h in normal medium (RPMI 1640, 10% FCS) with or without the specified treatment. At the end of the incubation period, LSECs were harvested and counted. LSECs (equal cell number for each sample) were incubated with 10 µM DCFH-DA (dissolved in DMSO) for 30 min at 37°C. After three washes with PBS (pH=7.0), the relative levels of fluorescence were quantified by flow cytometry (FACScan, Becton Dickinson, USA).

### Preparation of cytoplasmic and nuclear protein

For immunoblot analysis, the extraction and isolation of nuclear protein were performed using the Nuclear and Cytoplasmic Protein Extraction Kit (Beyotime, China) according to the manufacturer's protocol. Briefly, after treatment of LSECs with the indicated compounds, the cells were washed with 1 mL of ice-cold PBS, then collected and centrifuged for 5 min at 1200 *g* at 4°C. The resulting pellet was dissolved with cytoplasmic protein extraction agent A supplemented with 1 mM phenylmethylsulfonyl fluoride (PMSF). After 5 s of vortexing, the tubes were incubated for 12 min on ice to promote lysis. Then, cytoplasmic protein extraction agent B was added, the sample was vortexed for 5 s and incubated on ice for 5 s. The samples were then centrifuged for 5 min at 15,000 *g* at 4°C and the supernatant was immediately frozen for further analysis. The pellet was resuspended in nuclear protein extraction agent supplemented with 1 mM PMSF. The sample was vortexed 15 times, 30 min each time followed by centrifugation at 15,000 *g* for 10 min at 4°C. Supernatants containing nuclear extracts were obtained.

### Statistical analysis

Student's *t*-test or one-way ANOVA were used for statistical analysis. All statistical analyses were performed using SPSS 19.0 (IBM Corp., USA). P<0.05 was considered statistically significant.

## Results

### GSTD treatment reduced apoptosis, protected cell viability, and reduced ROS and MDA content of H_2_O_2_-exposed LSECs

**Figure 1.**
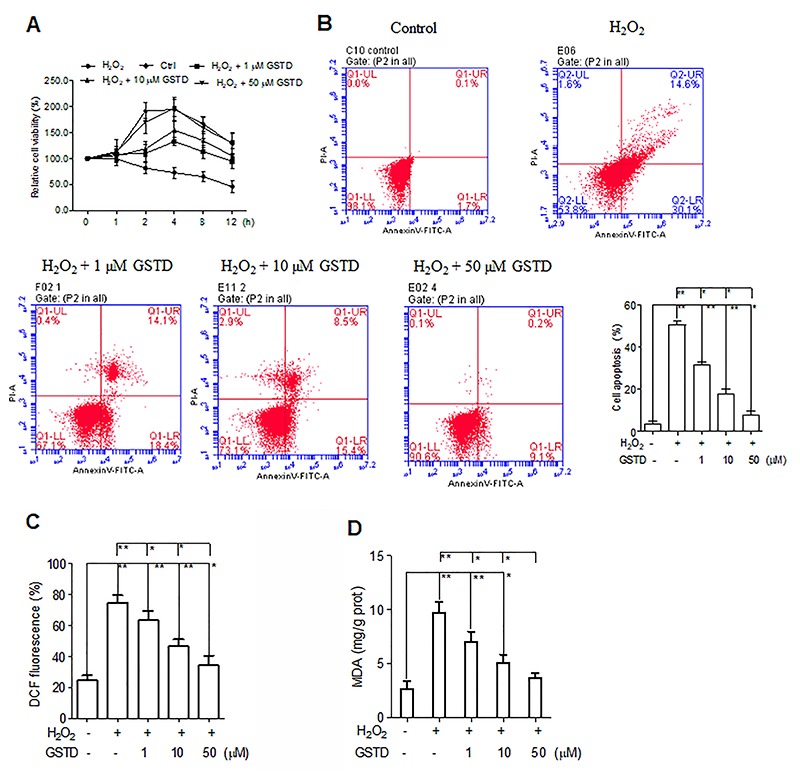
Gastrodin (GSTD) treatment reduced cell apoptosis and protected cell viability in H_2_O_2_-exposed liver sinusoidal endothelial cells (LSECs). *A*, Cell viability. Control (Ctrl) refers to those free of H_2_O_2_ and GSTD culture. *B,* Cell apoptosis using flow cytometric analysis. *C*, Reactive oxygen species (ROS) generation evaluated by the oxidation of H2DCF-DA to DCF. *D*, Malondialdehyde (MDA) content using the thiobarbituric assay. Data are reported as means±SD. *P<0.05, **P<0.01 (Student's *t*-test).

As shown in Figure 1A and B, exposure to 1 mM H_2_O_2_ significantly induced apoptosis and reduced viability of LSECs. Treatment with 1, 10, or 50 µM GSTD significantly reduced apoptosis by 57.5% (P<0.05), 66.8% (P<0.05), or 77.5% (P<0.01), respectively, *vs* untreated cells. The protective effects of GSTD on cell viability and apoptosis were dose-dependent. We detected ROS accumulation in these cells using a fluorescence assay with H2DCF-DA as the probe. ROS levels of LSECs were significantly elevated after H_2_O_2_ exposure (Figure 1C). Treatment of H_2_O_2_-exposed LSECs with 1, 10, or 50 µM GSTD decreased ROS levels by 11.3%, 28.0%, and 40.4% (P<0.05), respectively, compared with untreated cells (Figure 1C). This observation suggested that GSTD had a protective role against oxidative stress induced by H_2_O_2_. This was further supported by the reduction of MDA content in H_2_O_2_-exposed LSECs treated with GSTD *vs* untreated cells (Figure 1D). This reduction was dose-dependent.

### GSTD treatment suppressed caspase-9 cleaved expression of H_2_O_2_-exposed LSECs

**Figure 2.**
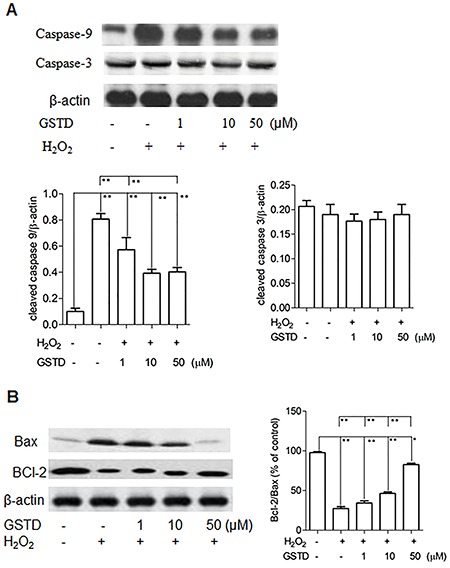
Gastrodin (GSTD) treatment suppressed caspase-9 pathway and maintained high Bcl-2/Bax ratio in H_2_O_2_-exposed liver sinusoidal endothelial cells (LSECs). *A*, Levels of cleaved caspase-9 and caspase-3 using Western blot. *B*, Levels of Bcl-2/Bax ratio. β-Actin was used as the internal control. Data are reported as means±SD. *P<0.05, **P<0.01; *A*, Student's *t*-test; *B*, one-way ANOVA test.

Because GSTD protected viability of H_2_O_2_-exposed LSECs by reducing apoptosis, we next examined the effects of GSTD on caspase-3 and caspase-9 cleaved expression. The results showed that caspase-9 cleaved expression was significantly enhanced by H_2_O_2_ exposure (P<0.01, Figure 2A). GSTD significantly suppressed H_2_O_2_-induced caspase-9 cleaved expression in a dose-dependent manner (P<0.01). H_2_O_2_ exposure and GSTD treatment had no effect on the expression of caspase-3 (P>0.05). Compelling evidence in the literature suggests that apoptosis is tightly regulated by the balance of a negative regulator, B-cell lymphoma-2 (Bcl-2), and a positive regulator, Bcl-2 associated X protein (Bax) (4). As shown in Figure 2B, Bax protein levels were increased and Bcl-2 protein levels decreased in LSECs by H_2_O_2_ exposure. GSTD treatment of these cells blocked the increase of Bax and decrease of Bcl-2, thus impeding the reduction of the Bcl2/Bax ratio induced by H_2_O_2_ exposure. Taken together, these results indicate that GSTD was able to reduce LSECs apoptosis induced by H_2_O_2_-exposure through suppression of the caspase-9 pathway and maintenance of a high Bcl2/Bax ratio.

### GSTD treatment induced HO-1 and Nrf2 upregulation in H_2_O_2_-exposed LSECs

**Figure 3.**
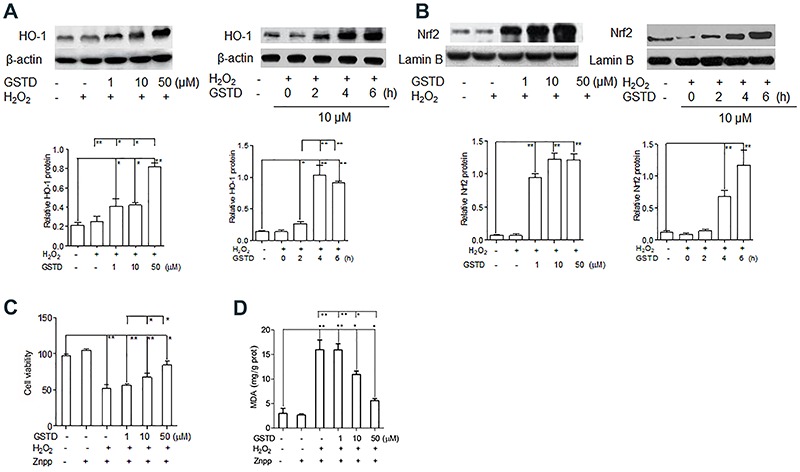
*A*, Heme oxygenase-1 (HO-1) expression and *B*, nuclear factor erythroid-related factor 2 (Nrf2) expression in H_2_O_2_-exposed liver sinusoidal endothelial cells (LSECs) induced by gastrodin (GSTD) treatment, using Western blot. *C*, Cell viability. *D*, Malondialdehyde (MDA) content assessed by the thiobarbituric assay. Data are reported as means±SD. *P<0.05, **P<0.01 (Student's *t*-test).

We speculated that the protective effects of GSTD treatment on cell viability was associated with the Nrf2/HO-1 pathway. Therefore, HO-1 and Nrf2 expression was evaluated. Western blot showed that HO-1 expression of H_2_O_2_-exposed LSECs was not significantly different from the normal control (P>0.05, Figure 3A). Treatment of these cells with 1, 10, or 50 µM GSTD significantly enhanced the HO-1 expression of H_2_O_2_-exposed LSECs by 64.0% (P<0.05), 68.2% (P<0.05), and 228.1% (P<0.01), respectively, compared with untreated cells (Figure 3A). The peak expression of HO-1 protein occurred 4 h after GSTD treatment. The expression of Nrf2 followed a similar pattern to that of HO-1 (Figure 3B). Nrf2 expression of H_2_O_2_-exposed LSECs was not significantly different from those of normal controls (P>0.05). GSTD treatment resulted in a significant increase of Nrf2 expression in H_2_O_2_-exposed LSECs, which was both dose- and time-dependent (P<0.05, P<0.01). To further confirm HO-1 involvement in the protection of cell viability, 10 µM Znpp, a specific inhibitor of HO-1, was added to the cell culture. The results showed that the addition of 10 µM Znpp completely abolished the protective effect of 1 µM GSTD treatment on LSEC viability. However, treatment with 10 or 50 µM GSTD showed protective effects to a certain degree on LSECs viability in the presence of 10 µM Znpp. The MDA content of LSECs treated with 1 µM GSTD was significantly increased in the presence of Znpp, with MDA content levels similar to LSECs without GSTD treatment. MDA content of LSECs treated with 10 or 50 µM GSTD in the presence of 10 µM Znpp were significantly lower than those of 1 µM GSTD-treated LSECs or untreated cells, indicating that 10 and 50 µM GSTD treatment maintained a certain protection for the oxidative injury induced by H_2_O_2_ exposure when HO-1 was inhibited. It was concluded that the HO-1 expression induced by GSTD treatment was directly involved in the protection of LSECs viability.

### GSTD treatment induced p38 mitogen-activated protein kinase phosphorylation to upregulate the HO-1/Nrf2 pathway

**Figure 4.**
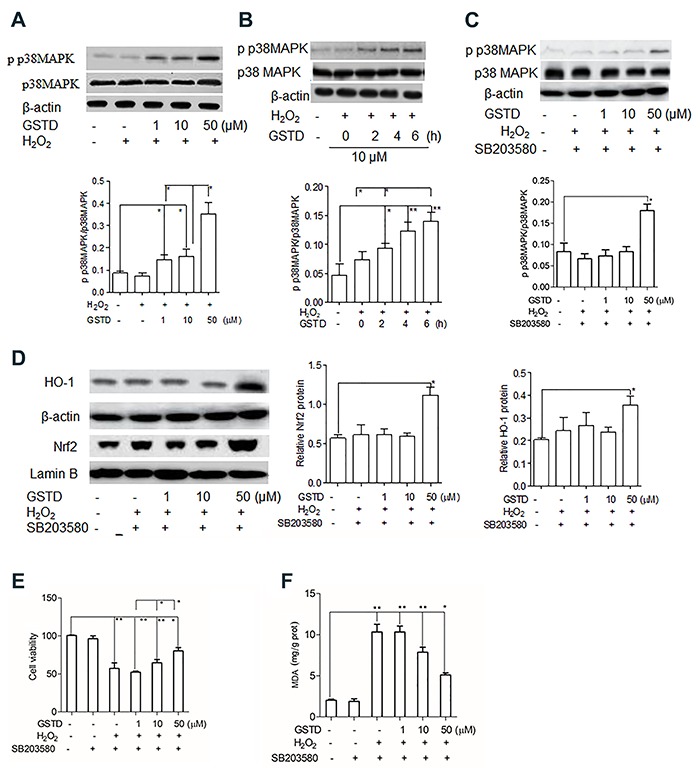
Gastrodin (GSTD)-induced heme oxygenase-1 (HO-1) and nuclear factor erythroid-related factor 2 (Nrf2) expression in liver sinusoidal endothelial cells (LSECs), *A*, Changes of p p38/p38 MAPK assayed using Western blot. *B*, p p38/p38 MAPK values under 10 µM GSTD treatment at different times. *C*, Effect of the inhibitor SB203580 on p p38/p38 MAPK. *D*, HO-1 and Nrf2 expression using Western blot. β-actin and lamin B were used as the internal standard to quantify HO-1 and Nrf2 expression, respectively. *E*, Cell viability. The absorbance at 450 nm was recorded. *F*, Malondialdehyde (MDA) content assessed by the thiobarbituric assay. Data are reported as means±SD. *P<0.05, **P<0.01 (Student's *t*-test).

To further elucidate the possible mechanism associated with GSTD-induced HO-1 and Nrf2 upregulation, levels of p38 mitogen-activated protein kinase (MAPK) and phosphorylated (p) p38 MAPK were examined. We found that p38 MAPK protein levels in GSTD-treated LSECs exposed to H_2_O_2_ were not significantly different from those of control and untreated cells (P>0.05, Figure 4A). However, levels of p p38 MAPK were significantly enhanced by GSTD treatment (P<0.05, P<0.01, Figure 4B and C), suggesting that the p p38 MAPK cascade was associated with HO-1 and Nrf2 expression. To confirm this, 2 µM SB203580, a specific inhibitor of p38 phosphorylation, was added to the culture in GSTD-treated LSECs. As shown in Figure 4D, the increase of HO-1 and Nrf2 expression induced by the treatment of 1 and 10 µM GSTD in H_2_O_2_-exposed cells was significantly inhibited by the addition of SB2033580 (P<0.05). In the presence of 2 µM SB203580, H_2_O_2_-exposed LSECs treated with 50 µM GSTD showed significantly higher HO-1 and Nrf2 expression than those in control and lower dose GSTD (1 and 10 µM) treatment (P<0.05). Incubated with the inhibitor, 10 and 50 µM GSTD treatment still showed significantly higher viability than those of 1 µM and without GSTD treatment (P<0.05, P<0.01, Figure 4E), although the viability of H_2_O_2_-exposed LSECs treated with 1 µM GSTD was not significantly different from those without GSTD treatment (SB203580 plus H_2_O_2_). In the presence of inhibitor, MDA content of H_2_O_2_-exposed LSECs treated with 1 µM GSTD was not significantly different from those without GSTD treatment (SB203580 plus H_2_O_2_), but was significantly higher than those treated with 10 µM GSTD. H_2_O_2_-exposed LSECs treated with 50 µM GSTD in the presence of SB203580 had significantly lower MDA content than those treated with 1 and 10 µM GSTD (P<0.05, P<0.01, Figure 4F).

## Discussion

Oxidative stress is a common pathological mechanism involved in several types of liver disease, including NAFLD, IR, drug- and non-drug-induced liver injury, and hepatic fibrosis ([Bibr B10] 11–12). Because the pharmacological action of GSTD is mainly associated with anti-inflammatory and antioxidant activities, we focused on looking into the antioxidant mechanisms of GSTD treatment in H_2_O_2_-treated LSECs.

Apoptosis in tissues is common under the physiological condition of stress and disease. In this study, we found that 1 mM H_2_O_2_ induced apoptosis and reduced viability of LSECs. The results are consistent with a previous study, which reported that cell survival of rat LESCs was remarkably reduced by H_2_O_2_ (24). Treatment with GSTD reduced H_2_O_2_-induced apoptosis and protected cell viability in a dose-dependent manner. When looking into the mechanism of apoptosis reduction by GSTD treatment, we found that GSTD suppressed H_2_O_2_-induced caspase-9 pathway activation and maintained a high Bcl2/Bax ratio. GSTD was previously reported to suppress 1-methyl-4-phenylpyridine (MPP^+^)-induced caspase-3 pathway activity in human dopaminergic SH-SY5Y cells (25). In the present study, the caspase-3 pathway was not affected by GSTD treatment or H_2_O_2_ exposure. The caspase-9 pathway rather than the caspase-3 pathway of LSECs may be sensitive to oxidative stress. H_2_O_2_-induced ROS accumulation is one of the most common reasons for cell death (26,27). Elevation of MDA levels is commonly used to indicate oxidative injury by ROS species. We found that GSTD treatment reduced ROS generation in H_2_O_2_-exposed LSECs in a dose-dependent manner. Similarly, the MDA content of GSTD-treated cells were significantly lower than untreated cells after H_2_O_2_ exposure. It was concluded that GSTD treatment reduced H_2_O_2_-induced oxidative injury and protected the viability of LSECs.

HO-1 catalyzes the oxidative cleavage of the α-mesocarbon of Fe-protoporphyrin-IX, yielding equimolar amounts of biliverdin-IXα, free divalent iron, and carbon monoxide (CO) (28). HO-1 can be induced by a variety of stimuli, most of which are linked by their ability to induce oxidative stress (29). HO-1 induction potentially confers protection against oxidative stress in a variety of experimental models, such as liver IR secondary to transplantation or hemorrhage/resuscitation (30,31). The human liver generates comparable amounts of the HO-1-derived products in both Kupffer cells and hepatocytes in the presence of sufficient substrates (32). However, there are no reports regarding their function and expression in LSECs.

After discovering that GSTD treatment provided protection against H_2_O_2_-induced oxidative stress, we investigated the effect of GSTD on HO-1 expression in H_2_O_2_-exposed LSECs. The results showed that HO-1 expression of H_2_O_2_-exposed LSECs was enhanced by GSTD treatment. When the catalytic function of HO-1 was inhibited by Znpp, the protective role of GSTD on viability became weaker and a higher MDA was observed compared with cells that did not receive Znpp, suggesting that HO-1 plays a key role in the defense of H_2_O_2_-induced oxidative stress in LSECs. These data indicate that GSTD exerted antioxidative effects on H_2_O_2_-exposed LSECs by inducing HO-1 protein expression. Interestingly, we observed that the HO-1 expression of H_2_O_2_-exposed LSECs was not statistically different from normal controls. HO-1 protein expression in LSECs seemed unresponsive to H_2_O_2_-induced stress. However, it should be noted that nearly 50% of LSECs were found to be apoptotic after exposure to H_2_O_2_. LSECs may be too sensitive to 1 mM H_2_O_2_ stress with the resulting low cell viability, which can give a misleading result on whether H_2_O_2_ stress is able to induce a change in HO-1 protein expression in LSECs.

Among the many mechanisms that regulate HO-1 protein expression, Nrf2 is a typical transcription factor that regulates an array of genes involved in detoxification and antioxidant defense. Recently, upregulation of Nrf2 induced by GSTD was found to alleviate MPP^+^-induced oxidative injury in SH-SY5Y cells (33). Nrf2 nuclear translocation and HO-1 protein expression were both induced by GSTD, ameliorating oxidative stress in a mouse model of NAFLD (6). Nrf2 expression was also evaluated. The pattern of detection of Nrf2 expression was similar to that of HO-1, as both were induced by GSTD treatment in H_2_O_2_-exposed LSECs. HO-1 upregulation by GSTD may occur at the transcriptional level, requiring Nrf2 regulation.

MAPK cascades have been shown to be involved in HO-1 activation. The synthesis of HO-1 protein and the activation and translocation of Nrf2 were reported to be regulated by p38 (34–36). The p38 cascade may protect Nrf2 from cytosolic degradation by facilitating nuclear translocation of the protein. Several studies showed that multiple inducers of HO-1 also activated protein phosphorylation-dependent signaling cascades, which ultimately depended on the transcription factors that regulate *HO-1* gene expression (37,38). We conducted the experiments designed to determine a possible role of MAPK pathways in regulating GSTD-induced HO-1 expression. The results showed that p38 was activated by GSTD treatment. The use of a specific inhibitor for p38 pathway, SB203580, confirmed the involvement of p38 in GSTD-induced HO-1 expression, Nrf2 nuclear translocation, and cell viability protection. In addition to MAPK cascades, it should be noted that GSTD may regulate HO-1 and Nrf2 expression via other pathways. For example, AMPK activation by GSTD was reported to increase the levels of Nrf2 phosphorylation and ameliorate oxidative stress/proinflammatory response in a mouse model of NAFLD (6). ERK1/2 phosphorylation induced by GSTD was shown to regulate Nrf2 expression and nuclear translocation in response to both neurotoxicity in SH-SY5Y cells and rat hippocampal neurons, and motor deficits and oxidative stress in a mouse model of Parkinson's disease (32,39,40). To fully understand the mechanism of GSTD regulation of HO-1 and Nrf2 expression in H_2_O_2_-exposed LESCs, more comprehensive investigation is needed.

Apart from antioxidative protection, GSTD may have prevented H_2_O_2_-induced cell death by anti-inflammatory action. Apoptosis of cells treated with 10 µM GSTD was significantly lower than those treated with 1 µM GSTD in LSECs exposed to H_2_O_2_. A more potent protection on cell viability was provided by the treatment with 10 µM GSTD. However, HO-1 and p p38 MAPK levels induced by 1 and 10 µM GSTD were not statistically different. Treatment with GSTD at 1 µM and 10 µM seemed to show similar potency in regulating HO-1 and p p38 MAPK expression. When HO-1 function was inhibited by Znpp or SB203850, a more prominent protection of 10 µM *vs* 1 µM GSTD treatment on cell viability was still observed. Therefore, the stronger protection of cell viability from 10 µM GSTD treatment in H_2_O_2_-exposed LSECs may be attributed to more potent anti-inflammatory activity.

In summary, this study demonstrated for the first time that GSTD treatment alleviated H_2_O_2_-induced oxidative stress in LSECs by reducing ROS, cell apoptosis, and MDA content. GSTD-induced HO-1 and Nrf2 protein expression was involved in protecting H_2_O_2_-induced oxidative injury in LSECs, which might be a common protective mechanism for liver oxidative detoxification regulated by p p38 MAPK. GSTD may be a protective agent against hepatic diseases associated with oxidative stress.
