# Erratum for: “Gastrodin induced HO-1 and Nrf2 up-regulation to alleviate H_2_O_2_-induced oxidative stress in mouse liver sinusoidal endothelial cells through p38 MAPK phosphorylation” [Braz J Med Biol Res (2018) 51(10): e7439]

**DOI:** 10.1590/1414-431X20197439erratum

**Published:** 2019-02-25

Hongbin Zhang https://orcid.org/0000-0001-7203-6297 ^1,2*^, Bo Yuan https://orcid.org/0000-0001-9210-8235 ^1*^, Hanfei Huang https://orcid.org/0000-0003-0927-5839 ^1^, Siming Qu https://orcid.org/0000-0001-8980-3114 ^1^, Shikun Yang https://orcid.org/0000-0001-9567-5622 ^1^ and Zhong Zeng https://orcid.org/0000-0003-3386-1299 ^1^

^1^Centre of Organ and Tissue Transplantation, the First Affiliated Hospital, Kunming Medical University, Kunming, Yunnan, China

^2^Department of Oncology, the First Affiliated Hospital, Kunming Medical University, Kunming, Yunnan, China

Correspondence: Zhong Zeng: <zzong9933@163.com>

^*^Hongbin Zhang and Bo Yuan are co-first authors.

**Erratum for:** Braz J Med Biol Res | doi: http://10.1590/1414-431X20187439

The Brazilian Journal of Medical and Biological Research would like to correct Figure 4 in the article “Gastrodin induced HO-1 and Nrf2 up-regulation to alleviate H_2_O_2_-induced oxidative stress in mouse liver sinusoidal endothelial cells through p38 MAPK phosphorylation” published incorrectly in volume 51 no. 10 (2018) <http://dx.doi.org/10.1590/1414-431X20187439>

The *β*-actin band in Figure 4A, 4C and 4D was erroneously presented in the preparation of the manuscript sent by the authors. The authors found the error after the publication of the paper and the correct Figure 4 is published below. The authors apologize to the readers and to the Brazilian Journal of Medical and Biological Research.

**Figure 4:**
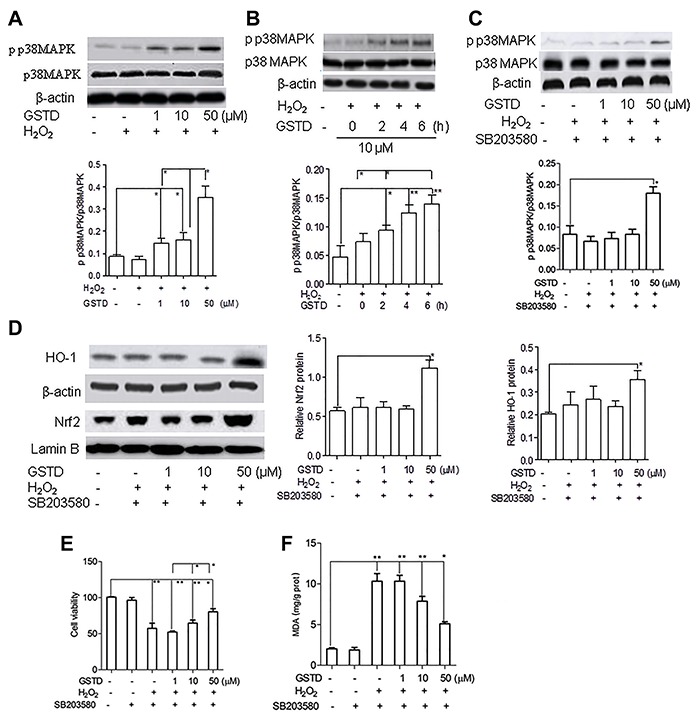
Gastrodin (GSTD)-induced heme oxygenase-1 (HO-1) and nuclear factor erythroid-related factor 2 (Nrf2) expression in liver sinusoidal endothelial cells (LSECs). *A*, Changes of p p38/p38 MAPK assayed using Western blot. *B,* p p38/p38 MAPK values under 10 µM GSTD treatment at different times. *C*, Effect of the inhibitor SB203580 on p p38/p38 MAPK. *D*, HO-1 and Nrf2 expression using Western blot. β-actin and lamin B were used as the internal standard to quantify HO-1 and Nrf2 expression, respectively. *E*, Cell viability. The absorbance at 450 nm was recorded. *F*, Malondialdehyde (MDA) content assessed by the thiobarbituric assay. Data are reported as means±SD. *P<0.05, **P<0.01 (Student's *t*-test).

